# Normal and cancer stem cells of the human female reproductive system

**DOI:** 10.1186/1477-7827-11-53

**Published:** 2013-06-19

**Authors:** Jacqueline López, Francisco J Valdez-Morales, Luis Benítez-Bribiesca, Marco Cerbón, Alejandro García Carrancá

**Affiliations:** 1 Programa de Doctorado en Ciencias Bioquímicas, Facultad de Química, Universidad Nacional Autónoma de México, Mexico City, Mexico; 2 Facultad de Química, Biología de la Reproducción, Universidad Nacional Autónoma de México, Mexico City, Mexico; 3 Unidad de Investigación Médica en Enfermedades Oncológicas, Hospital de Oncología, Centro Médico Nacional Siglo XXI IMSS, Mexico City, Mexico; 4 Unidad de Investigación Biomédica en Cáncer, Instituto de Investigaciones Biomédicas, Universidad Nacional Autónoma de México, San Fernando No. 22, Col. Sección XVI, Tlalpan 14080, Mexico City, Mexico; 5 División de Investigación Básica, Instituto Nacional de Cancerología, Secretaría de Salud, San Fernando No. 22, Col. Sección XVI, Tlalpan 14080, Mexico City, Mexico

**Keywords:** Stem cells, Cancer stem cells, Human, Female, Reproductive biology

## Abstract

The female reproductive system (FRS) has a great capacity for regeneration. The existence of somatic stem cells (SSC) that are likely to reside in distinct tissue compartments of the FRS is anticipated. Normal SSC are capable of regenerating themselves, produce a progeny of cells that differentiate and maintain tissue architecture and functional characteristics, and respond to homeostatic controls. Among those SSC of the FRS that have been identified are: a) undifferentiated cells capable of differentiating into thecal cells and synthesizing hormones upon transplantation, b) ovarian surface epithelium stem cells, mitotically responsive to ovulation, c) uterine endometrial and myometrial cells, as clonogenic epithelial and stromal cells, and d) epithelial and mesenchymal cells with self-renewal capacity and multipotential from cervical tissues. Importantly, these cells are believed to significantly contribute to the development of different pathologies and tumors of the FRS.

It is now widely accepted that cancer stem cells (CSC) are at the origin of many tumors. They are capable of regenerating themselves, produce a progeny that will differentiate aberrantly and do not respond adequately to homeostatic controls. Several cell surface antigens such as CD44, CD117, CD133 and MYD88 have been used to isolate ovarian cancer stem cells. Clonogenic epithelial and stromal endometrial and myometrial cells have been found in normal and cancer tissues, as side population, label-retaining cells, and CD146/PDGF-R beta-positive cells with stem-like features. In summary, here we describe a number of studies supporting the existence of somatic stem cells in the normal tissues and cancer stem cells in tumors of the human female reproductive system.

## Background

Somatic stem cells (SSC) share three common features; i) generate identical cells retaining this capacity over long periods (referred as long-term self-renewal), ii) produce a progeny that differentiates into mature cells exhibiting specialized functions, iii) respond to homeostatic controls regulating decision to self renew or produce differentiating progenitors. Contrary, cancer stem cells (CSC) although self-renew, generate a progeny that differentiates albeit aberrantly, and fail to properly respond to homeostatic controls. CSCs can be defined experimentally by their ability to recapitulate a continuously growing tumor.

**Figure 1:**
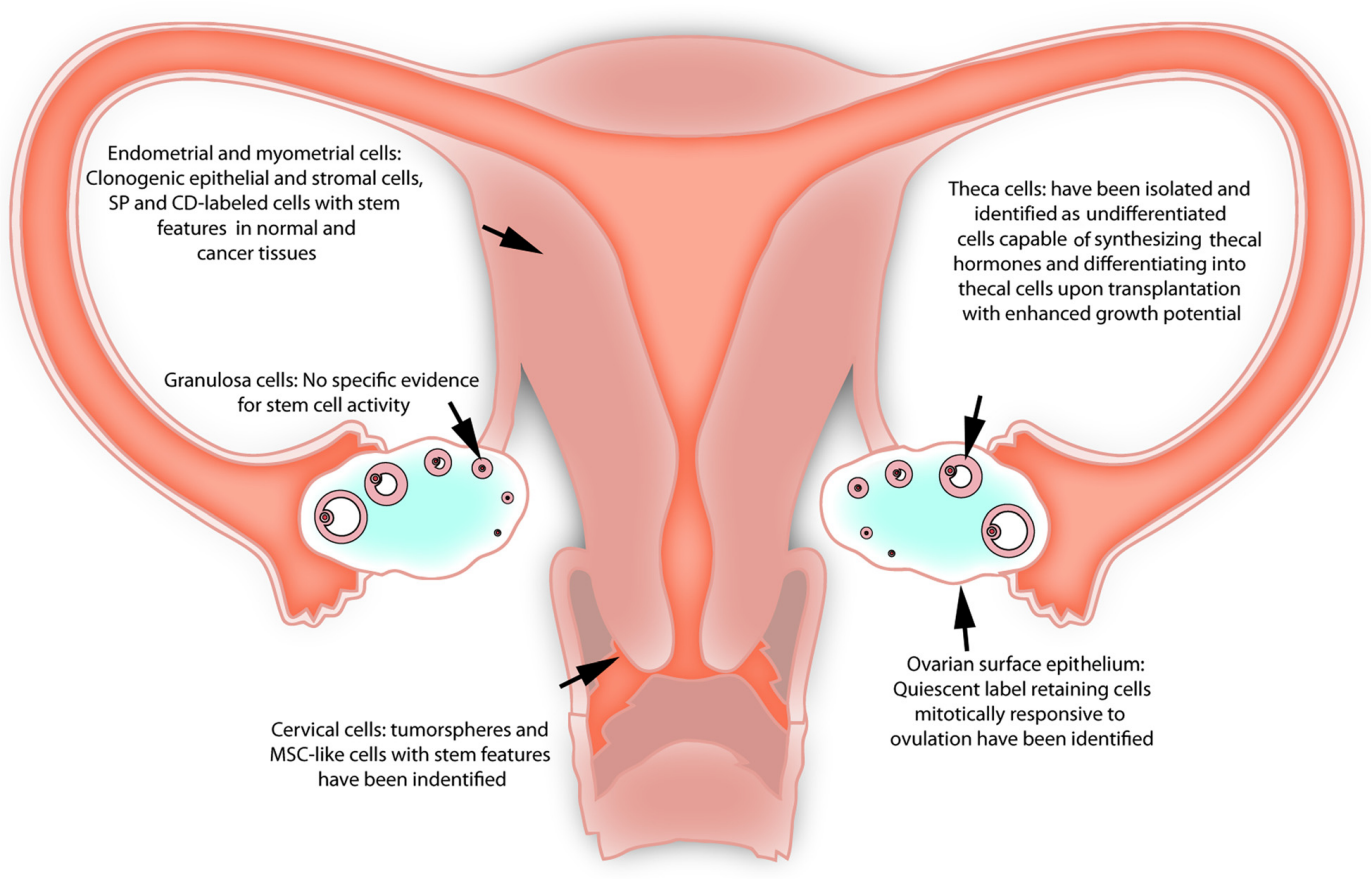
**Summary of current evidences supporting the existence of stem cells in the female reproductive system.** The existence of stem/progenitor cells is indicated in primary functional parts of the reproductive system, including ovary, uterus and cervix. Abbreviations: CD = Cluster of differentiation, MSC = Mesenchymal stem cell, SP = Side population.

Existence of stem cells within distinct tissue compartments of the FRS is well documented, as the contribution of CSCs in the development of different neoplasias (Figure 1). Experimental strategies for isolation and identification of cancer stem cells, as well as major tumor types originating within the FRS together with genetic mutations and clinical treatments are shown (Table 1). We present evidences based in an extensive description of markers expression and functional assays (Table 2) supporting existence of both normal and cancer stem cells in the human FRS, as well as their role in the normal physiology and gynecological pathologies.

**Table 1:** Major tumor types in gynaecological cancer; genetic mutations and clinical treatments

**Organ**	**Common tumor type**	**Mutations**	**Treatment**
**Ovary**[12]	**Ovarian epithelial carcinoma**	Loss of heterozygosity of PTEN, P53, K-ras, HNPCC mutations. BRCA1 and 2 inactivating mutation. ABCG2 overexpression, β-Catenin expression	**Early stage:** Ovarian surgery. Rarely detected at an early stage, because there is no definitive marker, but a combined immunoassay with six markers [leptin, prolactin, osteopontin, insulin-like growth factor II, macrophage inhibitory factor and CA 125]. In addition, the use of kallikrein or protease M and osteopontin are helpful
	Endometrioid, Serous, Clear cells, Transitional, Mucinous and Undifferentiated carcinoma		
	**Endometriosis related tumor**		**Advanced stage:** Platinum based chemotherapy and adjuvant radiotherapy
	Adenofibroma, Tubal intraepithelial carcinoma		**Novel Agents:** Antiangiogenic agents, Tyrosine kinase, SRC and PARP-1 inhibitors.
	**Germ cell tumor**		
**Uterus**[28]	**Endometrial carcinoma type I** [estrogen] and **II** [no estrogen related], **Adenocarcinoma Sarcoma**	PTEN inactivation, K-ras, P53 and P16 mutations, Microsatellite instability, HER-2/neu overexpression	**Early stage:** Hysterectomy, bilateral salpingoophorectomy and pelvic and periaortic lymph node dissection. Hormonal therapy with Progesterone in the form of an intrauterine device [IUD], cyclic progesterone or Megace
	**Myometrium cancer**		**Advanced and/or recurrent stage:** Chemotherapy with doxorubicin, paclitaxel and the platinum agents
	Leiomyoma [Commonly benign smooth muscle tumor]		Adjuvant radiation. Hormonal Therapy with medroxyprogesterone
	Endometrial cancer in advanced stage with myometrial invasion		**Novel Agents:** Ephotilones, Mammalian target of Rapamycin [mTOR] Inhibitors, Angiogenesis inhibitors. Myomectomy, and/or advanced stage treatments
**Cervix**[29]	**Cervical carcinoma**	K-ras mutations Carbonic anhydrase	**Early stage:** Laparotomic lymphadenectomy Radical trachelectomy, Hysterectomy. Adjuvant and neoadjuvant platinum-based chemotherapy. Local radiotherapy
	Squamous-cell, adenocarcinoma, adenosquamous and small-cell carcinoma	IX [CAIX], CAXII, hypoxia-inducible factor 1α, and VEGF upregulation	**Advanced and/or recurrent stage:** Adjuvant Chemotherapy
		Deregulated expression of E6 and E7 viral oncogenes	**Novel Agents:**Genome wide expression arrays, MiRNAs, Hyperbaric oxygenation for tumour sensitisation to radiotherapy.

The early or advanced stage depends on the extent of local spread [shown by the tumour stage and tumour size], nodal status and histological subtype. The grading is based on the WHO International Histological Classification of Tumours and Classification of Diseases for Oncology.

**Table 2:** Markers of cancer stem cells of the female reproductive system

**Tumor type**	**Marker**	**References**
**Ovarian cancer**	SP+	Szoket, 2006 [2]
	Clonogenic cells	Bapat, 2005 [3]
	CD44+, CD117+	Zhang, 2008 [4]
	CD133+	Curley, 2009 [5]
		Baba, 2009 [6]
		Ferrandina, 2008 [7]
		Kusumbe, 2009 [9]
	CD44+, MYD88+	Alvero, 2009 [8]
**Uterine cancer**	Clonogenic cells	Hubbard, 2009 [24]
	SP+	Friel, 2008 [25]
		Kato, 2010 [26]
**Cervical cancer**	p63 y CK17	eliminated
	Nanog, Musashi-1, Nucleostemin	Ye, 2008 [30]
	Spheres, ALDH1, CD44	Li, 2011 [33]
	Spheres-CD49f	Li, 2011 [33]
	Spheres	López, 2012 [32]
	ALDH1	Gu, 2011 [34]
	CD44	

Abbreviations: *CD*, Cluster of differentiation, *LRC*, Label-retaining cell, *SP*, Side population, *PDGF-Rβ*, PDGF-receptor β, *ALDH*, Aldehyde dehydrogenase, *MSC*, mesenchymal stem cell.

### Normal ovarian and cancer stem cells

#### Functional assays

Isolation of SC from the theca and ovarian surface epithelium (OSE) has been possible recently. Thecal stem cells were obtained after dissociating newborn mice ovaries and growing them in serum-free germline stem cell media [1]. Nonadherent anchorage-independent spheres exhibited appropriate gene profiles, compatible with theca cells that differentiate into early precursors and steroidogenic cells in a stepwise manner after treatment with serum, luteinizing hormone, and paracrine factors from granulosa cells, and later secreted androstenedione. At each step these cells displayed appropriate gene expression profiles and morphological features and achieved a mature morphology when coculture with isolated granulosa cells. In addition, they colonized exclusively the ovarian interstitium and the theca layer of follicles when transplanted into ovaries of recipient animals [1].

A population of label-retaining cells (LRC) residing in the coelomic epithelium and exhibiting quiescence, *in vivo* functional response to hormonal stimulus, and enhanced *in vitro* colony formation have been identified as candidate for somatic stem/progenitor cells of the mouse ovary [2].

Existence of ovarian CSCs is supported by identification and isolation of tumorigenic sphere-forming clones from ascites of patients with epithelial ovarian cancer [3]. Immunohistological evidence suggested differentiation along epithelial, granulosa, and germ cell lineages. Independent clones showed an ability to form spheroids and multicellular colonies in soft agar correlating with tumorigenicity. Xenografted tumors could be serially passaged through at least three generations *in vivo*, indicating their capacity to self-renew.

#### Markers

Ovarian CSCs were found to form tumors faster and with less inoculums, when injected into the dorsal fat pad of nude mice. Müllerian-inhibiting substance was able to reduce the growth of these cells *in vitro*. Surface proteins such as c-Kit, CD44 and CD133 have been associated with ovarian cancer cells with stem-like phenotype [4-8]. Expression of CD133-1 and CD133-2, which were detected in ovarian carcinomas, was also observed in normal ovaries. CD133^+^ ovarian tumor cells were characterized by a higher proliferative potential and clonogenic efficiency than negative cells [7]. CD133^-^ cells from cancer cell lines, primary tumors and ascitic fluid of ovarian cancer patients were shown to be tumorigenic [6]. CD133^+^ cells derived from ovarian tumors were capable of self-renewal and were associated with increased tumor aggression in xenografts. Furthermore, they identified that epigenetic deregulation of CD133 may be associated with transformation [6]. Using *in vivo* serial transplantations, contribution to establishment of tumor vasculature of these cells was demonstrated [9]. Other studies showed that CD133+ ALDH + coexpressing cells had greater tumor initiating capacity in ovarian cancer cell line and primary human ovarian tumors [10,11]. Moreover, CD133,CD117, CD44 and CD24 markers could be used as CSC markers alone or in combination to identify distinct FRS CSC population. However, it is relevant to establish if the markers expressed are functionally related to each other, and their clinical implications.

In the other hand, it has been previously reported that several markers are also expressed in normal stem cells or even in other tissues, which implies the need of further studies to develop therapeutic targets and delimitate their activity as possible clinical treatments.

A study of ovarian serous adenocarcinomas identified a population of tumorigenic self-renewing ovarian CSCs that can grow as sphere-forming clusters under nonadherent conditions [4]. When xenografted, as few as 100 spheroid-dissociated cells allowed full recapitulation of the original tumor, whereas >1 × 10^5^ unselected cells remained nontumorigenic. Enhanced chemoresistance to cisplatin or paclitaxel and up regulation of stem cell markers (including Bmi-1, Stem Cell Factor, Notch-1, Nanog, Nestin, ABCG2, and Oct-4) were further established. Immunostaining showed significant up regulation of CD44 and stem cell factor receptor c-Kit [4].

A majority (71%) of 31 ovarian cancer samples analyzed expressed a complex pattern of CD44 splice variants. CD44S and CD44-9v were common features of epithelial ovarian cancer cells, although no association between CD44 variants expression and clinical stage, residual disease, age, histology, grade, or survival was observed, suggesting other factors may be more important in determining clinical behavior [12].

CD44^+^, MYD88^+^ cells from ascites and solid tumors have been characterized by constitutive nuclear factor kappa beta (NF-κβ) activity, cytokine and chemokine production, high capacity for repair, chemoresistance to conventional chemotherapies, resistance to TNF-α-mediated apoptosis, capacity to form spheroids in suspension, and the ability to recapitulate *in vivo* the original tumor [8]. The same research group identified bipotent CD44^+^ CD34^−^ cells in ovarian cancer and demonstrated that, in addition to being capable of tumor regeneration, these cells also contribute to tumor vascularization by a mechanism that involves inhibitor of kappa-kinase beta (IκK-β). Aldehyde dehydrogenase1 (ALDH1) did not appear to be co-expressed with CD44, CD117 and CD133. Furthermore, reduced ALDH1 expression was associated with malignant transformation in ovarian cancer.

### Normal uterine and cancer stem cells

#### Normal endometrial stem cells

Multiple endometrial stem cells (ESC) including epithelial, mesenchymal and endothelial cells may contribute to rapid endometrial regeneration following menstruation.

#### Markers

Endometrial mesenchymal stem cells (MSCs) are prospectively isolated as CD146^+^, PDGF-Rβ^+^ (platelet-derived growth factor receptor beta) cells and are found in both basalis and functionalis as perivascular cells. Endometrial epithelial progenitor cells (EPCs) have been detected as SP cells, expressing several endothelial cell markers and differentiating into epithelial, stromal and endothelial cells. Investigating ESC biology is crucial to understanding normal endometrial physiology and determining their roles in endometrial proliferative diseases [13-15].

#### Functional assays

Human epithelial and stromal cells from endometrial tissues showed approximately 0.15% of epithelial (0.01% forming large CFUs and 0.14% forming small CFUs) and 1.25% of the stromal cell populations are clonogenic (0.02% large CFUs and 1.23% small CFUs) [13-15]. Large epithelial colonies show reactivity for alpha(6)-integrin (CD49f). All colonies contain fibroblasts expressing stromal markers, and the highest clonogenicity of stromal cells is observed in the proliferative stage, whereas peak clonogenicity of epithelial cells is found in the secretory stage. Interestingly, clonogenicity within the stromal and epithelial cell fractions do not differ between actively and inactively menstruating women, suggesting that ovarian-derived steroid hormones do not maintain the clonogenic potential of uterine epithelial and stromal tissues [13].

Subsequent studies on stromal CFUs indicate that individual large CFUs had substantial self-renewal activity *in vitro*, undergoing serial subcloning 2.9 and 3.3 times, respectively, whereas small CFUs serially cloned 0.5 only one time [16]. On the other hand, large epithelial and stromal CFU stem cell-like progenitors have high proliferative potential and undergo 34 and 30 population doublings before senescence or transformation [16].

Single large stromal CFUs derived from freshly isolated endometrial tissue underwent multilineage differentiation into four mesodermal lineages when cultured under appropriate conditions including smooth muscle cells, adipocytes, chondrocytes and osteoblasts [16]. Stromal clones expressed MSC markers ITGB1 (CD29), CD44, NT5E (CD73), THY1 (CD90), ENG (CD105), PDGF-Rβ (CD140B), MCAM (CD146) but not endothelial or hemopoietic markers. Adult human endometrium contains rare epithelial progenitors and MSCs, likely responsible for its immense regenerative capacity, which may also play critical roles in the development of endometriosis and EC [16,17]. According to this, endometrial cell clones derived from *in vitro* cultured and purified stromal cells display characteristic stem cell features including clonality, long-term culturing properties, multilineage differentiation potential, expression of CD146, CD105, CD90, CD73, Msi-1, Notch1, and Sox2, and absence of CD34 and CD14 expression [18]. This finding is supported by more extensive differentiation studies in which rare (1.5%) CD146^+^, PDGF-Rβ^+^ stromal cells could be induced to differentiate into osteocytes, chondrocytes, myocytes, and adipocytes [19,20]. CD146^+^, PDGF-Rβ^+^ cells expressed typical MSC surface markers, CD29, CD44, CD73, CD90 and CD105 and were negative for hemopoietic and endothelial markers. These cells were located perivascularly in both functionalis and basalis layers of human endometrium.

SP cells have been identified in fresh endometrium isolates and short-term cultures of human endometrial cells, with high variability among subjects, although higher numbers were found in the menstrual and proliferative stages, with around 0.0–5.1% of cells in normal human endometrium constituting this fraction [16]. Cervello *et al.* characterized the SP corresponding to the stromal and epithelial compartments using endometrial SP (ESP) gene signatures, immunophenotyping and characteristic telomerase pattern. They demonstrated functional capability of ESP to develop human endometrium after subcutaneous injection in non-obese diabetes/severe combined immunodeficiency (NOD/SCID) mice [17].

A medium specific for endothelial cell culture enabled SP cells to proliferate and differentiate into various types of endometrial cells including glandular epithelial, stromal and endothelial cells *in vitro*, whereas in the same medium, endometrial main population (MP) cells differentiated into only stromal cells. Furthermore, SP cells, but not MP cells, reconstituted organized endometrial tissue with well-delineated glandular structures when transplanted under the kidney capsule of severely immunodeficient mice. Notably, SP cells generated endothelial cells that migrated into the mouse kidney parenchyma and formed mature blood vessels [21]. Together these data indicate that SP cells both *in vitro* and *in vivo* produce endometrial epithelial and stromal cells; however, the hierarchical relationship between SP cells, clonogenic cells, CD146^+^, PDGF-Rβ^+^ cells, and tissue-reconstituting cells remains to be elucidated.

### Normal myometrial stem cells

#### Functional assays

SP cells were isolated from the myometrium (myoSP) of patients undergoing hysterectomy [22]. myoSP resided in quiescent cells, and myometrial cell markers were under expressed or missing. These cells could proliferate and eventually differentiate into mature myometrial cells *in vitro* only under low oxygen concentration. Although the main population expressed myo (myoMP) and displayed mature myometrial phenotypes before and after *in vitro* cultivation, only myoSP, not myoMP, generated functional human myometrial tissues efficiently when transplanted into the uteri of severely immunodeficient mice. Finally, myoSP were multipotent and made to differentiate into osteocytes and adipocytes *in vitro* under the appropriate differentiation-inducing conditions. Thus, myoSP exhibited phenotypic and functional characteristics of myometrial stem cells. Study of myoSP will improve the understanding of myometrial physiology and the pathogenesis of myometrium-derived diseases such as leiomyoma. myoSP may also represent a novel source of biological material that could be used in the reconstruction of not only the human uterus but also other organs as well [22].

#### Markers

Human and murine myometrial progenitors have been characterized by surface markers and found CD31^+^, CD34^+^, CD44^+^, CD117^+^, Stro-1^+^ and Sca-1^+^. These cells can differentiate *in vitro* into a number of mesodermal (smooth and skeletal muscle, osteoblasts and adipocytes) as well as epidermal lineages (all neural lineages). Importantly, when injected into animal models of muscular disease, this population can regenerate new muscle fibers and promote functional muscular recovery. Moreover, these cells can regenerate the uterine lining after wound healing, reconstructing the uterine muscular architecture and forming new vessels both *in vitro* and *in vivo.* These results strongly suggest that a resident population of myometrial cells can functionally behave like myometrial stem cells.

### Endometrial cancer stem cells

#### Functional assays

In a study of a uterine carcinosarcoma-derived cell line, colony-initiating cells grew for >50 serial passages and were composed of cells with columnar, small epithelial, moderately sized or large epithelial like, malignant tumor giant and spindle-shaped morphologies, similar to those found in the original cell line. These highly proliferative clonal cells expressed immunohistochemical and molecular markers consistent with their parental tissue and recapitulated the tumor phenotype *in vitro*[23].

Isolated endometrial carcinoma cells, when transplanted under the kidney capsule of immunocompromised mice in serial dilution 2 × 10^6^ −1 × 10^4^ cells, generated tumors in 8/9 samples with morphologies similar to the parent tumors. These tumors recapitulated cytokeratin, vimentin, estrogen receptor alpha, and progesterone receptor expression of the parent tumor. Clonally derived endometrial carcinoma cells also expressed the self-renewal genes BMI-1, Nanog, and Sox-2. Isolated cells from primary tumors were serially transplanted three to five times in NOD/SCID mice, showing self-renewal *in vivo*[24,25].

A study has examined several cell lines and four high-grade EC samples for the presence of SP cells [25]. In the AN3CA and Ishikawa, but not the SKUT-2 and HEC-1 cell lines, rare SP cells were detected (3.4%) demonstrating CSCs traits, including slow growth, as evidenced by a higher percentage of cells in G1, and their capacity to initiate tumors in NOD/SCID mice when injected subcutaneously [25]. Furthermore, the HEC-1-A SP population was showed to be clonogenic and self-renewed in the serial cloning assay and initiated larger tumors than the non-SP population. Interestingly, HEC-1-A SP cells produced tumors comprising epithelial tumor cells and vimentin-, α-SMA- and collagen III-expressing stromal cells, indicating that an epithelial to mesenchymal transition had occurred during cancer progression of the SP cell-initiated tumors *in vivo*[26].

In a study examining 113 patient samples covering the full spectrum of EC, primary tumor samples exhibited a variable degree of immunoreactivity for CD133/1 (1.3 − 62.6%). Dissociated bulky tumors formed sphere-like structures, maintained CD133 expression and could be propagated for up to 12 weeks. CD133^+^ cells purified from endometrioid adenocarcinomas were resistant to cisplatin- and paclitaxel-induced cytotoxicity and expressed a peculiar gene signature consisting of high levels of matrix metalloproteases, interleukin-8, CD44, and CXCR4. When serially transplanted into NOD/SCID mice, CD133^+^ cells were capable of initiating tumor formation and recovering the phenotype of the original tumor [27].

#### Markers

ALDH1 was detected in a small population of endometrial tumor cells that were less mature. ALDH1-positive cells were more tumorigenic, resistant to anticancer agents, and more invasive than ALDH1 negative or low cells. Clinically, high-level of ALDH1 was correlated with lymphatic invasion, recurrence, and poor prognosis of patients. ALDH1 is a candidate CSCs marker for uterine endometrioid adenocarcinoma [28]. Msi-1 was immunolocalized to single epithelial cells and small clusters of stromal cells in endometrial, endometriotic and endometrial carcinoma tissue specimens. Msi-1^+^ cells were mainly found in the basalis in the proliferative stage of the menstrual cycle, suggesting their possible stem/progenitor cell function. Stromal Msi-1^+^ cells were not found in a perivascular location, although some were in a periglandular region, a similar location to some stromal LRCs in mouse endometrium [15]. A large proportion of endometriotic glands expressed Msi-1^+^. Immunofluorescence microscopy revealed colocalization of Msi-1 with its molecular target Notch-1 and telomerase.

In endometrium, MSCA-1, a bone marrow-derived MSC surface marker identified as tissue nonspecific alkaline phosphatase (TNAP), is expressed at intermediate levels on CD146^+^ cells and at high levels in the luminal space of glandular epithelia. In conclusion, human endometrium also harbors a rare population with MSC and fibroblast properties that can be partially purified as a CD146^+^, PDGF-Rβ^+^ population. A higher proportion of the rare these cells differentiate compared to unfractionated fibroblasts, and individual CFU are truly multipotent as their progeny differentiate into multiple mesodermal lineages. This suggests that a hierarchy exists in the MSC-fibroblast lineage. Clearly this needs further molecular, genetic and *in vivo* characterization.

### Cervical cancer stem cells

#### Markers

Although limited, evidence supporting the existence of stem-like cells in cervical tumors is convincing. They have been identified in the uterine cervical epithelium as P63 and CK17-positive cells in cervical intraepithelial neoplasia (CIN) grades I - III. In all cases, P63 was found strongly expressed in the basal layer of the lesions. The distribution pattern and marker profile of reserve cells along the adult human endocervical canal was studied and two subpopulations of reserve cells were found: a CK17-positive subpopulation in the lower part of the cervical canal with a progenitor cell function for the squamous and columnar epitheliums, and a subpopulation of CK17-negative reserve cells with a progenitor cell function only for columnar cells [29].

Ye *et al.* examined the expression of Nanog, Nucleostemin (NS) and Musashi1 (Msi1) in cervical epithelial lesions and in cervical carcinomas and assessed their association with several prognostic variables. There was an association between expression of these three proteins and the severity of epithelial changes; levels were significantly higher in cervical squamous cell carcinoma (CSCC) compared with CIN, and with normal cervical epithelia. High expression of these proteins may be involved in carcinogenesis of the cervix and progression to cervical carcinoma. However, there was no positive correlation between expression levels and clinical pathological prognostic factors [30]. The expression of other markers as PSCA, PIWIL1 and TBX2 was evaluated in CSCC and normal adjacent cervix. In general, expression rates were higher in cancer and associated with invasion. Also, expression of *SOX2* was evaluated in normal and pathologic cervical tissues, and in cervical cancer tumorspheres and differentiated cells. While80% of CIN III or CSCC expressed Sox2 protein, compared with only 25% of normal cervix, CSCC grades II and III showed relatively higher intensity of *SOX2* staining compared with that of squamous carcinoma I. Also, *SOX2* was strongly expressed in primary tumorspheres derived from fresh cervical cancer tissues, but was never or seldom detected in differentiated cells. Additionally, it was found that exogenous SOX2 could promote both cell proliferation and growth, and enhanced tumor formation in nude mouse. Contrary, Cantz *et al.* were unable to detect significant levels of *OCT4* mRNA or protein in HeLa cells, and found that *OCT4* promoter region is highly methylated in these cells [31]. These authors argue that reports of *OCT4* expression in this and other cancer cell lines could in reality be attributed to the expression of six *OCT4* pseudogenes or to misinterpretation of background signals. Expression of ALDH1 in cervical carcinoma was evaluated and it was found that 23/89 invasive squamous carcinomas and 4/20 adenocarcinomas exhibited immunoreactivity to ALDH1and that cervical carcinoma cells had low CD133 expression, similar to found by Lopez *et al.*[32].

#### Functional assays

Epithelial-mesenchymal transition (EMT) can endow cells with stem cell-like characteristics. Li *et al.* induced EMT in breast cancer MCF7 and CC HeLa cells with expression of Twist, a key transcriptional factor for this transition. They also found that expression of ALDH1 and CD44 were significantly elevated in Twist-over expressing cells, and that β-catenin and Akt pathways were activated. This study suggests that this activation is critical for the maintenance of EMT, and that targeting β-catenin and Akt pathways can suppress EMT-associated stem cell-like properties [33].

A CSC population from primary carcinoma of the cervix uteri was identified. Eight of 19 tumor-derived cultures encompassed CSC capable of self-renewal and extensive proliferation as clonal non-adherent spherical clusters. Spheroids were identified as CD44^+^ CK17^+^, and while only 48% of sphere-forming cells were inhibited by doxorubicin, 78% of non-sphere forming cells were inhibited. Xenoengraftment of 1 × 10^5^ dissociated spheroid cells allowed full recapitulation of the original tumor, whereas the same amount of non-adherent spheroid selection remained non-tumorigenic. They found that spheroid cells were CD34 negative, as shown by Lopez and colleagues [32].

Gu *et al.* isolated Sphere-forming cells (SFC) from HeLa and SiHa cell lines and found they were tumorigenic with 1 × 10^4^ cells. They further demonstrated that HeLa-SFC expressed a higher level of the HPV oncogene E6, compared with that of parental HeLa cells. Silencing of E6 inhibited HeLa-SFC sphere formation and cell growth. They found all three isoforms of the transformation growth factor-β (TGF-β) were significantly down regulated while the leukemia-inhibitory factor remained unchanged. This suggests that E6 silencing exerts a specific effect on the expression of *TGF-β*[34].

Lopez *et al.* characterized a self-renewing subpopulation of CSC among four cancer-derived cell lines, HeLa, SiHa, Ca Ski, and C-4 I, and found that these express the CSC markers characteristic of the FRS including *CD44, ITGB1* (CD29), *PSCA, NT5E* (CD73), *ENG* (CD105), *MYC* (c-Myc), *PCGF4* (BMI-1), and *ABCG2.* Other epithelial CSC markers found included *ITGB6, ALCAM* (CD166), and *MET* (c-Met) [32]. Interestingly, components of the double-strand break DNA repair machinery and genes involved in the metabolism of reactive oxygen species were also up-regulated and indeed, dose-dependent radiation assays indicated that CSC-enriched populations exhibit increased resistance to ionizing radiation. CSC enriched as spheroids highly expressed CD49f and could generate reproducible tumor phenotypes in immunodeficient nu-nu mice and could be propagated serially. Injection of 1 × 10^3^ dissociated cells from spheroids induced tumors in the majority of animals, as opposed to injection of 1 × 10^5^ cells grown as monolayer. In addition, EMT transition-associated markers were found highly expressed in spheroid cells.

Together these results suggest that cervix CSCs participate in carcinogenesis of this tissue and these cells may be potential therapeutic target molecules for cervical cancers; however, this is a new area under investigation and many questions remain to be answered.

## Conclusions

Stem cells play a pivotal role in the physiology of the normal FRS and are likely to be involved in the response of these tissues to injury and disease. Many studies have provided strong evidence for the existence of SSCs in the human endometrium and ovary. However, stem cell biology of the human FRS is still in its infancy, and although surface markers for prospective isolation of human endometrial colony-forming cells have been identified, there remains a need to identify definitive markers for more selective isolation and enrichment of stem cells from all tissues and organs of the FRS. Complete characterization of these stem/progenitor cells will improve our understanding of the mechanisms supporting physiological regeneration of the FRS. Additionally, further investigations are needed to evaluate the clinical correlation between CSC population features, poor prognosis and progression free survival. Moreover, is important to establish the functional relationship between markers, since it is known that some are also widely expressed and shared by normal tissues and stem cells. Therapeutic approaches that directly target these molecules may be limited and more concerns about specific effects need to be considered. Animal transgenic and xenografts model systems also need to be implemented in order to examine the hallmark characteristics of FRS stem cells and shared by all stem cells, i.e., potential for self-renewal, lineage differentiation and homeostatic control. Such studies will enhance our understanding of ovarian, uterine and cervix cancer and may prove helpful in the treatment of these conditions.

## Competing interests

The authors declare that they have no competing interests.

## Authors’ contributions

JLG and FJVM participated in the acquisition of information and prepared the first draft of the manuscript. All authors analyzed the information and approved the final manuscript. Financial support for research was provided by Instituto de Ciencia y Tecnología del Gobierno del Distrito Federal (ICyT-GDF; GI/PIFUTP08-142 to AGC), Consejo Nacional de Ciencia y Tecnología-México (CONACYT-México grants No. 80338 to FJVM and No. 127822 to AGC), Programa de Apoyo a Proyectos de Investigación e Innovación Tecnológica de la Universidad Nacional Autónoma de México (PAPIIT-UNAM; IN226408 to AGC). All authors read and approved the final manuscript.
